# Polarization-Multiplexed Dynamic Light Scattering: Characterizing Rotational Diffusion and Shape of Optically Anisotropic Particles

**DOI:** 10.1021/acs.analchem.5c08209

**Published:** 2026-04-02

**Authors:** Lukas Grabenwarter, Miguel Spuch-Calvar, Patricia Taladriz-Blanco, Christian Moitzi, Sandor Balog

**Affiliations:** † 272858 Anton Paar GmbH, Anton-Paar-Str. 20, 8054 Graz, Austria; ‡ CINBIO, Universidade de Vigo, Campus Universitario Lagoas Marcosende, 36310 Vigo, Spain; § Adolphe Merkle Institute, 311305 University of Fribourg, 1700 Fribourg, Switzerland; ∥ National Center of Competence in Research Bio-Inspired Materials, University of Fribourg, 1700 Fribourg, Switzerland

## Abstract

Rotational diffusion and shape analysis of optically anisotropic particles, such as the length and aspect ratio, is widely considered to be beyond the capabilities of standard batch-mode single-angle dynamic light scattering, which, unlike polarized and depolarized dynamic light scattering, does not benefit from multiangle and polarization-selective detection. Most desktop instruments lack these features; therefore, they are considered incapable of providing information about rotational diffusion and particle shape. Here we demonstrate that one can characterize rotational diffusion and the shape of particles with optical anisotropy without the use of any polarization filters, even at a single angle. By using a conventional desktop instrument with forward scattering low-angle detection, we demonstrate our approach by characterizing the principal dimensions and aspect ratios of gold nanostars, gold nanorods, and hematite spindles. Our approach opens up new possibilities that were previously inaccessible to users of standard dynamic light scattering.

## Graphical abstract

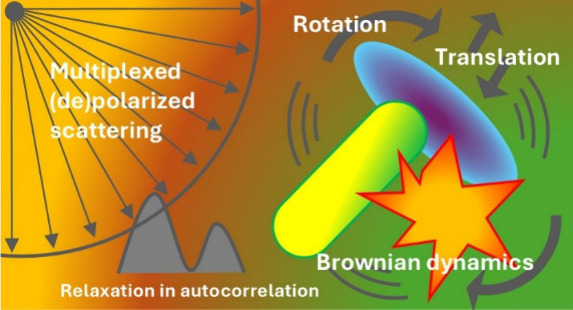

Dynamic light scattering (DLS) is among the most frequently used experimental techniques to characterize colloidal particles and soft nanomaterials.^1,2^ DLS is an in situ, noninvasive, contactless technique that is widely applied to characterize the solvated particle size and viscoelasticity of complex soft materials.^3^ DLS is known for its affordability, rapidity, and experimental simplicity, providing access to a time scale that ranges from submicroseconds to seconds, and a length scale spanning the nanoscale to the microscale. Optical anisotropy can be observed in various colloidal soft-matter systems, including metallic, polymeric/plastic (semicrystalline and crystalline particles), and diverse oxide particles. A compilation of such particle systems and their benefits is provided in the Supporting Information of the article by Bossert et al.^4^

Although DLS has been used to study anisotropic particles, previous work relied entirely on polarization-resolved vv/vh detection. To our knowledge, conventional single-angle, unpolarized DLS has never been used to extract rotational diffusion or the shape of optically anisotropic particles.

Optically anisotropic particles exhibit orientation-dependent extinction cross sections, meaning their absorption and scattering of a polarized laser beam vary with their alignment relative to the incident polarization vector. A colorful evidence of this phenomenon is given by the characteristic double-peak feature of the UV–vis extinction spectra of, e.g., gold nanorod dispersions,^5^ where one extinction peak corresponds to the transversal mode of localized surface plasmon resonance (representative of particle width), and the other to the longitudinal mode (representative of particle length).^6^

Optical anisotropy leads to depolarized light scattering (Figure 1), which enables the analysis of rotational Brownian motion and the extraction of the corresponding rotational diffusion coefficient.^7–9^ Light scattered from optically anisotropic particles, oriented randomly in dispersions, will not be polarized anymore but will contain many different polarizations. As illustrated in Figure 1, the polarization of the scattered light is a function of particle orientation (ϕ). Generally, the polarization vector of the scattered light (**P**) and the polarization vector of the laser’s electric field (**E**) are not parallel. The angle φ between the two vectors is a function of particle orientation:

1 $$\varphi =\cos^{-1}\frac{\alpha_{∥}\;\cos^{2}\phi +\alpha_{⊥}\;\sin^{2}\phi }{\sqrt{{\alpha_{∥}}^{2}\;\cos^{2}\phi +{\alpha_{⊥}}^{2}\;\sin^{2}\phi }}$$

where α_∥_ is the polarizability along the long axis and α_⊥_is the polarizability perpendicular to the long axis of an axisymmetric particle. Optical anisotropy arises when α_∥_ ≠ α_⊥_ (Figure 1b), but when α_∥_ = α_⊥_, the scattered light stays fully polarized.

**Figure 1:**
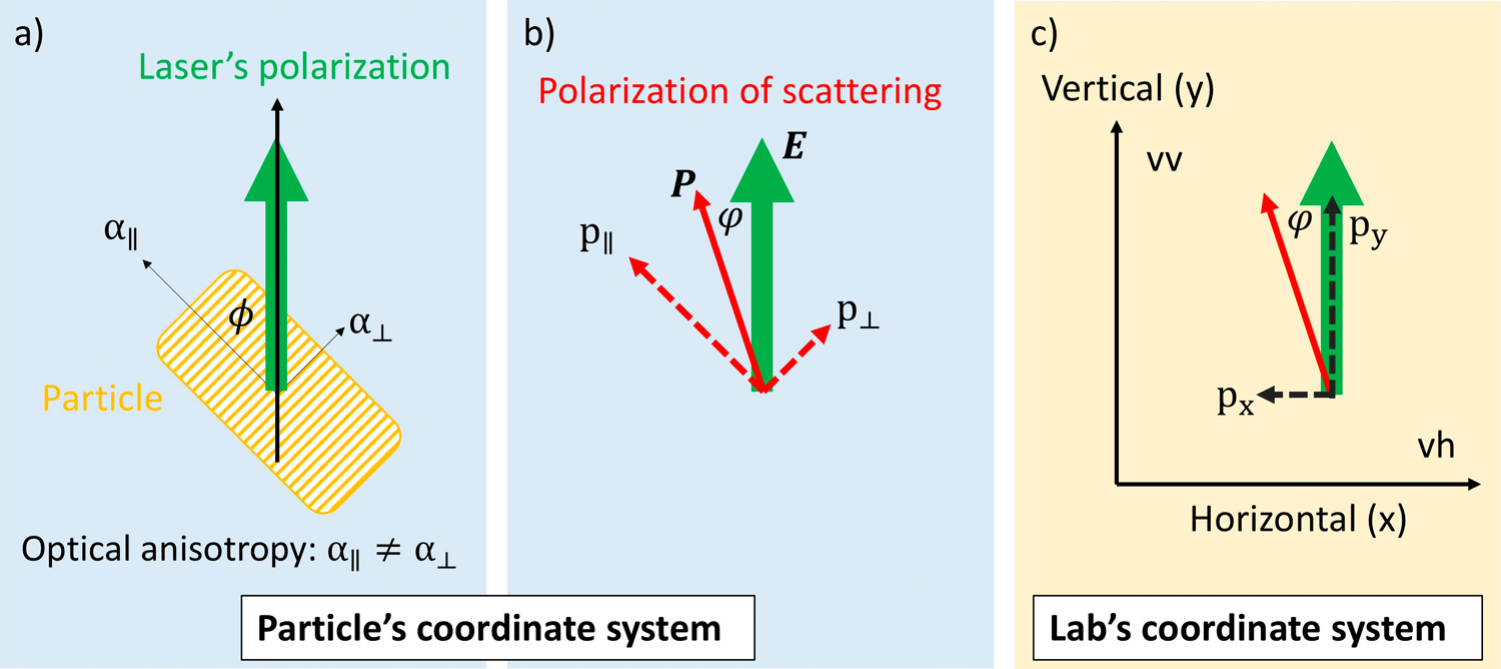
Origin of depolarized single scattering illustrated in two dimensions with a particle of axial symmetry. (a) Optical anisotropy (anisotropic polarizability) may be decomposed in the coordinate system of the particle: polarizability along the long axis (α_∥_) and perpendicular to the long axis (α_⊥_). If these two are unequal, the particle exhibits optical anisotropy. (b) As a consequence, the polarization vector (*p*_∥_ = α_∥_ *E* cos ϕ and *p*_⊥_ = α_⊥_ *E* sin ϕ) is not parallel to the laser’s polarization, and the scattered light becomes depolarized compared to the laser. The angle φ between the laser’s polarization and the polarization vector is a function of particle orientation (ϕ in (a)) and varies as the particle rotates. In the absence of optical anisotropy (α_∥_ = α_⊥_), the polarization of scattering is always parallel to the polarization of the laser, independent of the particle orientation: φ = 0 and *p*_*x*_ = 0. (c) Depolarization of the scattered light may be decomposed into two polarization modes that are parallel (vv: vertical–vertical, *p*_*y*_) and perpendicular (vh: vertical–horizontal, *p*_*x*_) to the axes of the lab’s coordinate system. Brownian motion will cause fluctuations in both components, which gives access to characterizing both translation and rotational diffusion. Particles dispersed in a solvent are oriented randomly, and thus, light scatters in many different polarizations. In classical DDLS, one uses the angle-dependent relaxation of the cross-polarized (vh) mode.

When a dynamic light scattering (DLS) instrument is equipped with a polarization filter (also known as an analyzer), both polarized (vv, vertical–vertical) and depolarized (vh, vertical–horizontal) components of the scattered light can be measured. The main reason for using depolarized dynamic light scattering (DDLS, also known as dynamic depolarized light scattering), where the depolarized component of scattered light is measured, is that it allows researchers to characterize rotational diffusion and, thus, characterize the shape of colloidal particles. A typical DDLS setup allows for the simultaneous determination of translational and rotational diffusion coefficients. In the vv configuration (Figure 1c), both the incident and detected scattered light are vertically polarized. This mode is primarily sensitive to intensity fluctuations caused by the translational Brownian motion of particles. The vh configuration (Figure 1c) involves vertically polarized incident light and detection of horizontally polarized scattered light. This mode is primarily sensitive to intensity fluctuations caused by the rotational Brownian motion of particles. These diffusion coefficients are linked to key particle geometry parameters (typically length and aspect ratio) through established theoretical models.^10,11^ DDLS can be a powerful tool for measuring both translational and rotational diffusion, allowing researchers to go beyond basic size estimation and gain insights into the particle shape.

An intuitive extension of DDLS is the so-called multipolarization (or partial depolarization) analysis, where an autocorrelation function is recorded with a polarization filter that is set at an intermediate angle between vv (φ = 0) and vh (φ = π/2), as illustrated in Figure 1c.^12–14^ Such an autocorrelation function may be written as a linear combination of two terms:

2 $$g_{1}\left(t,\varphi \right)=y\left(\varphi \right)e^{-\Gamma_{1}t}+\left[1-y\left(\varphi \right)\right]e^{-\left(\Gamma_{1}+\Gamma_{2}\right)t}$$

where Γ_1_ = *q* ^2^ *D*_T_ and Γ_2_ = 6*D*_R_, in which *D*_T_ and *D*_R_ are the translational and rotational diffusion coefficients and *q* = (4π/λ*n*) sin­(θ/2) is the momentum transfer, where θ is the scattering angle, λ is the wavelength of the laser, and *n* is the refractive index of the suspension. The weighting factor 0 ≤ *y*(φ) ≤ 1 is polarization-dependent, where φ is the angle between the filter and the polarization vector of the laser. Equation 2 describes how the detected scattering becomes a polarizer-angle-dependent linear combination of the vv and vh polarization modes. When the analyzer is rotated (φ), the weighting between vv (bimodal: translation + rotation) and vh (unimodal: rotation-dominated) changes accordingly. Thus, the observed relaxation reflects the controlled mixture of the two polarization channels. In the case of cylindrical (axis-symmetric) particles, *y*(φ) is expressed as a function of particle shape and polarizability:^14^

3 $$y\left(\varphi \right)=\frac{p_{1}\left(\varphi \right)}{{p_{1}\left(\varphi \right)+p}_{2}\left(\varphi \right)}$$

where *p*_1_(φ) = (2α_⊥_ + α_∥_)^2^ cos^2^ φ *P*(*q*) and *p*_2_(φ) = (α_⊥_ – α_∥_)^2^(3 + cos^2^ φ)/45, in which α_∥_ and α_⊥_are the optical polarizabilities parallel and perpendicular to the long axis of the cylinder and *P*(*q*) is the particle form factor averaged over particle orientation. Equation 3 gives the weighting factor used in eq 2. At φ = π/2, eq 2 gives *y* = 0, corresponding to pure vh. At φ = 0, however, *y* < 1 because the vv channel is intrinsically bimodal, so the weighting cannot reach unity.

It is worthwhile to recognize—and this study is based on that recognition—that standard DLS instruments without polarization filters are in fact true polarization multiplexing instruments that simultaneously detect all polarization states scattered from a particle. Accordingly, the correlation function may be written as an average over the angle of polarization (Figure 1, eq 1):

4 $$g_{1}\left(t\right)=\frac{1}{\pi }\int_{0}^{\pi }g_{1}\left(t,\varphi \right)\;d\varphi$$

Equation 4 boils down to evaluating the integral only on *y*(φ):

5 $$\left\langle y\right\rangle =\frac{1}{\pi }\int_{0}^{\pi }y\left(\varphi \right)\;d\varphi$$

which is readily obtained in closed form:

6 $$\left\langle y\right\rangle =\frac{\omega \left(1-\sqrt{3}\sqrt{\frac{{\left(\alpha_{∥}-\alpha_{⊥}\right)}^{2}}{4{\left(\alpha_{∥}-\alpha_{⊥}\right)}^{2}+\omega }}\right)}{{\left(\alpha_{∥}-\alpha_{⊥}\right)}^{2}+\omega }$$

in which ω = 45­(α_∥_ + 2α_⊥_)^2^ *P*(*q*), where α_∥_ and α_⊥_ are the axis-parallel and -perpendicular polarizabilities and *P*(*q*) is the particle form factor. In the absence of optical anisotropy (α_∥_ = α_⊥_), ⟨*y*⟩ = 1. In this case, there is no depolarized component, as expected, and only translational diffusion is responsible for the fluctuations in the scattering intensity. When α_∥_ ≠ α_⊥_, ⟨*y*⟩ < 1. With eq 6, we may express the polarization-multiplexed autocorrelation function as follows:

7 $$g_{1}\left(t\right)=\left\langle y\right\rangle e^{-\Gamma_{1}t}+\left[1-\left\langle y\right\rangle \right]e^{-\left(\Gamma_{1}+\Gamma_{2}\right)t}$$

Therefore, the polarization-multiplexed autocorrelation function retains the characteristic bimodal form and contains information about the rotational diffusion of optically anisotropic particles. This offers two key advantages to the experimentalists: (1) it eliminates the need for installing and aligning polarization filters, whether on a goniometer or at fixed scattering angles, and (2) it enables light detection with a significantly higher signal-to-noise ratio than conventional filter-based setups, thereby reducing the laser power requirements typically needed for vh-mode measurements. Thus, what initially appears to be a limitation of standard DLS (its lack of polarization filtering) can in fact be viewed as an advantage. By inherently collecting light across all polarization states, such systems effectively perform polarization multiplexing, enabling access to both translational and rotational dynamics. To further substantiate our point, we recorded and analyzed the intensity autocorrelation functions of dispersions of gold nanostars (GNS), hematite spindles (HES), and commercial gold nanorods (GNR). The particles are described in detail in the Supporting Information, along with representative transmission electron microscopy (TEM) images. Their dimensions estimated through TEM image analyses are summarized in Table 1.

**Table 1:** Particle Dimensions Estimated through TEM Image Analyses^a^

particle	length (nm)	aspect ratio
gold nanostar (GNS)	96 ± 9.5	–
hematite spindle (HES)	482 ± 100	6.5 ± 0.7
gold nanorod (GNR)	32 ± 5.5	2.5 ± 0.6

a Data are reported as mean ± STD.

To record the autocorrelation functions, we used a conventional desktop DLS instrument (Anton Paar Litesizer DLS 500, λ = 658 nm, maximum laser power of 40 mW). (The autocorrelation functions are available upon request from the corresponding author.) DLS instruments construct the so-called intensity autocorrelation function *g*_2_(*t*), while the theories address the so-called electric field autocorrelation function *g*_1_(*t*). The two are brought together by the Siegert relation: *g*_2_(*t*) = α + β|*g*_1_(*t*)|^2^, where the baseline α ≈ 1 and amplitude β ≈ 1 are frequently regarded as adjustable parameters when regressing experimental data points. We recorded the samples in the so-called batch-mode DLS, that is, without subjecting the samples to any purification and separation technique. All measurements were performed in rectangular polystyrene cuvettes at room temperature using forward scattering, with acquisition times of 1 min (GNS), 5 min (HES), and 3 min (GNR), and at least 15 successful runs for each sample. The setup scheme is shown in Figure SI-4. We employed a low-angle forward scattering detection geometry with θ = 15°. This scattering angle was chosen because the particle form factor remains close to unity (*P*(*q*) ≈ 1), allowing a less biased quantification of translational diffusion while facilitating the separation of translational and rotational dynamics. At low scattering angles, translational diffusion is probed over larger length scales compared to side or backscattering, resulting in a slower decay of the autocorrelation function, which enhances the resolvability of the relaxation modes described in eq 7. Ensembles of autocorrelation functions, as recorded, are shown in Figure 2.

**Figure 2:**
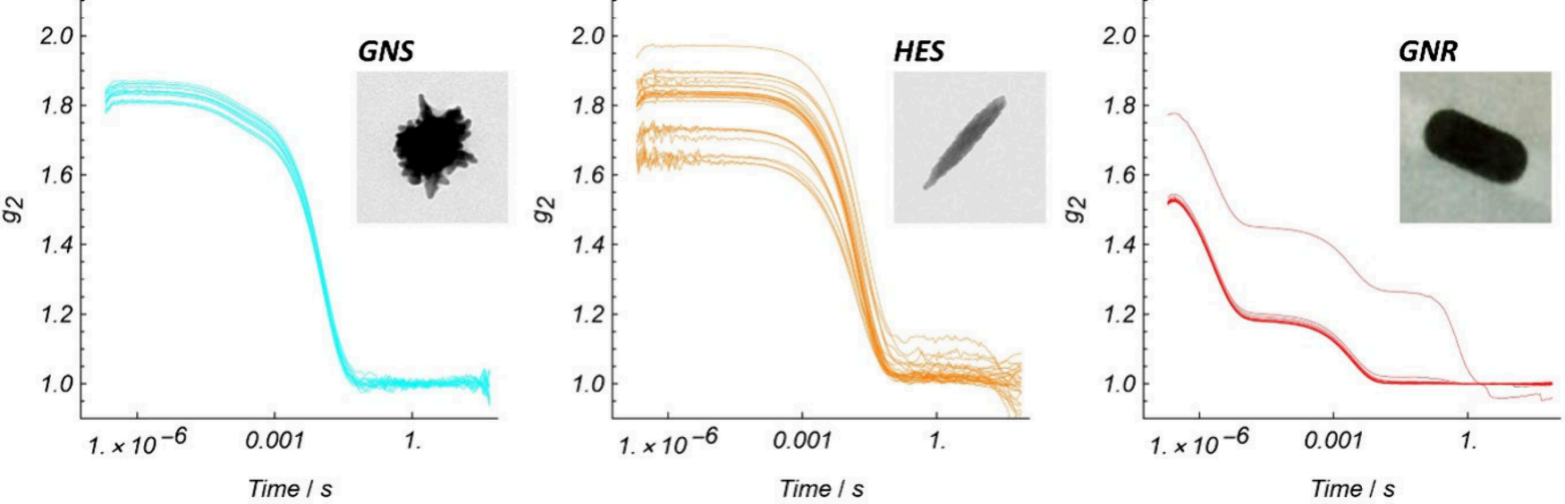
Autocorrelation functions of particles (Table 1) as recorded by our DLS instrument. The particle form factor *P*(*q*) modulates the polarization-angle weighting *y*(φ) and thus ⟨*y*⟩, with higher *P*(*q*) increasing the relative translational contribution. Although lower *P*(*q*) at larger scattering angles enhances the depolarized fraction, it also increases *q* ^2^ and accelerates translational relaxation, risking overlap with rotational decay. Consequently, optimal separability of the two modes is achieved at low-*Q* forward angles, as used in our experiments. Each particle system exhibited variability in volume, shape, length, and aspect ratio (Table 1). Complete TEM figures with scale bars are available in the Supporting Information.

There are variations in the amplitude and baseline of the raw autocorrelation functions (Figure 2). Such variability is common in batch-mode DLS because transient events, for example, particle number fluctuations in the scattering volume, can raise the baseline and slow the apparent decay of the autocorrelation function compared to stable, steady-state measurements. To mitigate the impact of this variability, autocorrelation functions were preprocessed and ranked. First, each autocorrelation function was cropped to the lag-time interval between 0.5 and 1s. Then, the baseline was estimated and subtracted, and the amplitude was normalized to one, and the autocorrelation function was further truncated to remove baseline points dominated by noise, by excluding correlation points when the signal-to-noise ratio (defined as the mean over the standard deviation) dropped below 1, as presented elsewhere.^15^ Second, we estimated the area under the autocorrelation curves numerically. This was done because transient dynamics affect the apparent relaxation of the autocorrelation function, and smaller areas out of a set of repetitions suggest stable, steady-state autocorrelations.^16,17^ We ranked the autocorrelation curves by their area and selected the three repetitions with the smallest areas. These were then averaged. We were able to model these autocorrelation functions using linear combinations of negative exponentials:

8 $$g_{2}\left(t\right)-1=\beta {\left(\overset{n}{\underset{i=1}{\sum }}a_{i}e^{-\Gamma_{i}t}\right)}^{2}$$

where β ≈ 1, *n* ≥ 2, *a*_*i*_ > 0, ∑_*i*=1_^*n*^ *a*_*i*_ = 1, and Γ_*i*+1_ > Γ_*i*_ > 0. We expected this simple model to be able to represent the modes relevant to translational and rotational Brownian dynamics (Figure 3 and Table 2).

**Figure 3:**
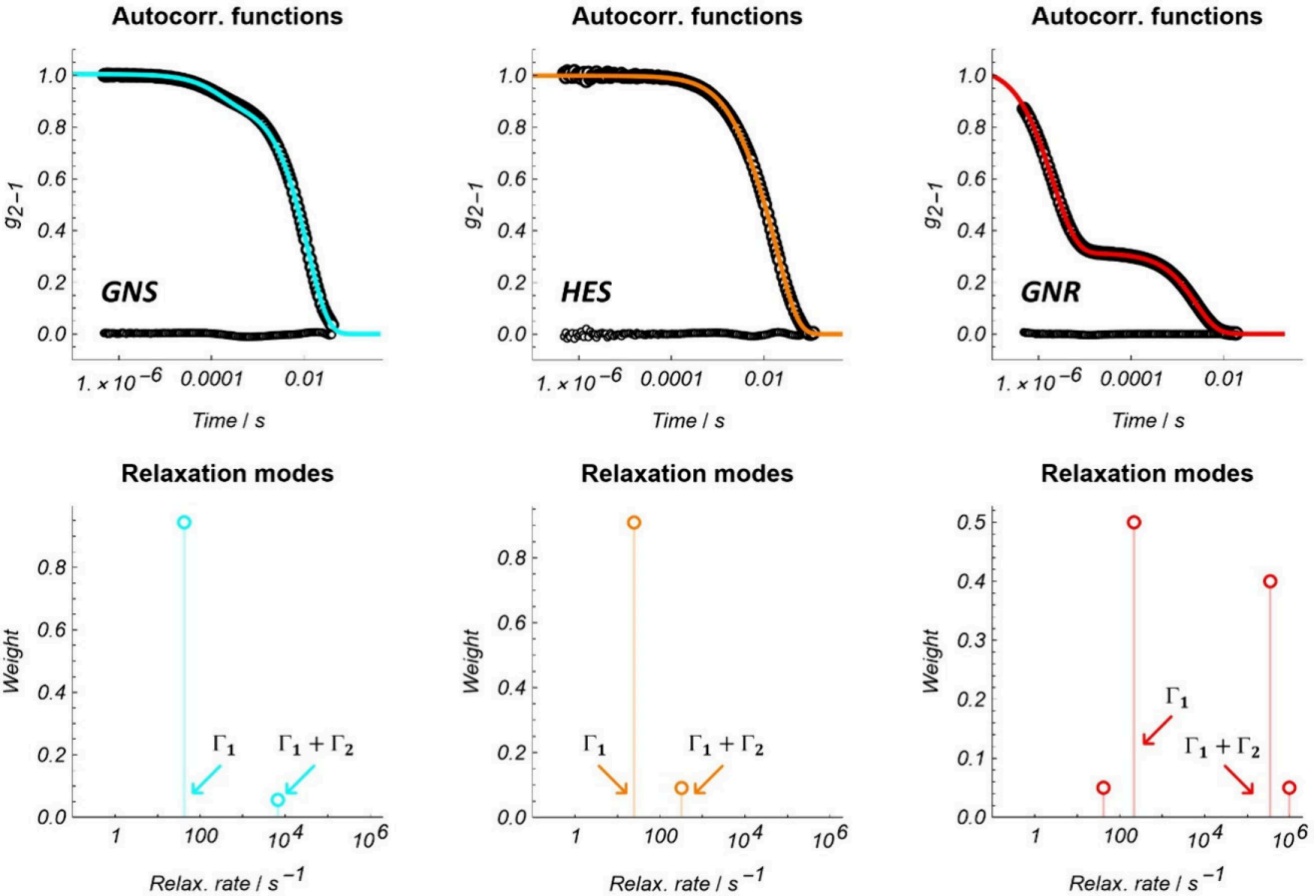
(top) Autocorrelation functions (symbols), the mathematical model (solid lines), and the corresponding residuals (symbols) of eq 8. (bottom) Modes corresponding to the autocorrelation functions. The modes indicated by the arrows were used for estimating diffusion coefficients: Γ_1_ = *q* ^2^ *D*_T_ and Γ_1_ + Γ_2_ = *q* ^2^ *D*_T_ + 6*D*_R_. The autocorrelation functions of the GNS and HES samples were well-described by two single modes. In contrast, the GNR sample required four modes, but we attribute the necessity of the two additional modes to sample heterogeneity. We used the two dominant modes, which accounted for 90% of the relative weight in the autocorrelation function and omitted the others.

**Table 2:** Modes of relaxation rates, diffusion coefficients, and particle dimensions estimated through polarization-multiplexed DLS^a^

particle type, shape model	relaxation rate (weight)	diffusion coefficients	dimensions^b^
gold nanostar (GNS), sphere	4.2 × 10^1^ s^–1^ (94%)	*D*_T_ = 3.6 × 10^–12^ m^2^ s^–1^	*d*_h,T_ = 115 nm
	6.7 × 10^3^ s^–1^ (6%)	*D*_R_ = 1.1 × 10^3^ rad^2^ s^–1^	*d*_h,R_ = 104 nm
hematite spindle (HES), spheroid	2.5 × 10^1^ s^–1^ (91%)	*D*_T_ = 2.2 × 10^–12^ m^2^ s^–1^	AR = 6
	3.2 × 10^2^ s^–1^ (9%)	*D*_R_ = 5.0 × 10^1^ rad^2^ s^–1^	*L* = 599 nm
gold nanorod (GNR), rod	*4.1 × 10^1^ s^–1^ (5%)*	*D*_T_ = 1.9 × 10^–11^ m^2^ s^–1^	cylinder: AR = 2.4, *L* = 40 nm
	2.2 × 10^2^ s^–1^ (50%)	*D*_R_ = 5.7 × 10^4^ rad^2^ s^–1^	sphero-cylinder: AR = 2.9, *L* = 43 nm
	3.4 × 10^5^ s^–1^ (40%)		
	*9.7 × 10^5^ s^–1^ (5%)*		

a The *italicized* relaxation modes for GNR are attributed to sample heterogeneities and were not used for estimating particle dimensions.

b *d*_h,T_ and *d*_h,R_ are the translational and rotational hydrodynamic diameters, respectively; AR is the aspect ratio; *L* is the length.

From the relaxation rates, we estimated the translational and rotational diffusion coefficients listed in Table 2. These coefficients were then used to quantify key particle geometry parameters through particle hydrodynamic models (Supporting Information, Hydrodynamic models). We used four shape models: (1) sphere for GNSs; (2) spheroid (ellipsoidal particle) for HESs, as done by Perrin^18,19^ and Koenig;^20^ and two types of rod for GNRs, namely, (3a) cylinders, as done by Ortega and García de la Torre,^21^ and (3b) sphero-cylinders (i.e., cylinders capped by hemispheres), as done by Fujita and co-workers.^22^ Martchenko et al.^10^ presented a succinct and informative summary of the experimentalist’s perspective on these shape models; therefore, we refrain from detailing them here. To estimate the diffusion coefficients, we used two relaxation rates for each particle system: one for Γ_1_ = *q* ^2^ *D*_T_ and the other for Γ_1_ + Γ_2_ = *q* ^2^ *D*_T_ + 6*D*_R_. We estimated the characteristic dimensions of the particles, listed in Table 2, using these hydrodynamic models. The results we obtained via the analysis of polarization-multiplexed autocorrelation functions are highly consistent with the particle shapes observed in electron microscopy (Table 1 and Figures SI-1–SI-3).

In summary, our study demonstrates that standard single-angle DLS instruments without polarization filters can characterize both rotational and translational diffusion, and therefore, for example, the shape of anisotropic particles. Indeed, by leveraging polarization multiplexing, the translational and rotational diffusion coefficients of gold nanostars, hematite spindles, and gold nanorods were extracted, and these coefficients were used to estimate particle dimensions and aspect ratios, which matched well the dimensions and shapes observed via electron microscopy. Therefore, standard DLS instruments are not strictly limited solely to size estimation, as they can resolve shape-related parameters of anisotropic particles, making shape analysis accessible to a broader user base. By expanding the capabilities of conventional DLS, we demonstrated that the absence of polarization filters is not always a drawback but may be an advantage, enabling the simultaneous detection of all polarization states and thereby improving signal quality.

With respect to accessible hydrodynamic sizes, defined by particle shape and dimensions such as length and aspect ratio, we do not observe any differences compared with standard DLS. The accuracy of our method is limited by the ability to resolve relaxation modes, and this constraint is the same as in conventional DLS. Resolving multiple modes in multimodal or polydisperse samples remains a numerical inversion problem: extracting relaxation-rate distributions from a noisy autocorrelation function. Our limitations therefore follow those of standard DLS. Any bias or uncertainty in the relaxation rates propagates through hydrodynamic shape models when estimating quantities such as length or aspect ratio. Most DLS instruments operate at fixed angles, typically providing forward, side, or backscattering detection. Forward scattering is optimal for separating rotational and translational relaxation contributions, since the translational rate scales with *q* ^2^ whereas the rotational rate is angle-independent. Forward angles do not automatically introduce bias, but the strong nonlinear dependence of light scattering (e.g., Rayleigh–Gans–Debye scattering) on the particle size causes the intensity and autocorrelation function to be weighted toward larger particles. As a result, even a small number of large particles, such as aggregates, can dominate the signal, requiring careful data collection and removal of outlier traces. This limitation is not specific to our method; standard DLS and DDLS are affected in the same way. The corresponding limitation for rotational diffusion is set by the lag times. Since the rotational mode is independent of scattering angle, its upper limit is set by the minimum accessible lag time: if the decay becomes too fast, the autocorrelation function will no longer capture the full relaxation.

Compared with classical DDLS, our polarization-multiplexed approach offers several practical and conceptual advantages. It eliminates the need for polarization optics entirely, avoiding the delicate vv/vh alignment required in DDLS and enabling measurements on any standard unpolarized DLS instrument. By collecting all scattered polarization states, the method achieves a substantially higher signal-to-noise ratio than the vh-filtered detection, which is typically weak and demands higher laser power. Moreover, both translational and rotational diffusion can be retrieved from a single scattering angle through mathematical separation, streamlining the experimental workflow. As a result, rotational-diffusion and shape analysis—traditionally limited to specialized DDLS setups—become broadly accessible on conventional benchtop DLS instruments. Regarding the general applicability, polarization-multiplexed DLS is versatile, and the principle broadly covers a wide range of optically anisotropic particle systems across diverse scientific disciplines, spanning from biological macromolecules to industrial samples, such as fibrous proteins, viruses, oxide and ceramic particles, and increasingly, microplastics and engineered polymer particles that exhibit optical anisotropy. Multiplexed DLS works on any standard DLS instrument, and thus, it greatly expands usability and accessibility. It can extract both translational and rotational diffusion without polarization filters, because standard detectors already collect all polarization states. Classical DDLS physically isolates the vh component, making it more convenient when translational and rotational modes overlap, but it requires dedicated optics and multiple scattering angles. In contrast, multiplexed DLS relies on the mathematical separation of modes; if the two relaxation rates overlap at a single angle, separation may become unreliable, in which case adding measurements at additional angles resolves the issue.

Nothing comes without a cost, however, and we must address the methodological boundaries and potential pitfalls of our approach. (1) It is important to note that DLS addresses single scattering, but multiple light scattering can also lead to depolarized light scattering, particularly in optically turbid and concentrated systems, even if they contain only homogeneous spherical particles.^23^ (2) In the context of single light scattering, depolarized scattering arises from optical anisotropy, which may result from (a) internal structural anisotropy, such as in semicrystalline materials, or (b) anisotropic particle geometry. Even morphologically spherical particles can exhibit measurable depolarized scattering if their internal structure is sufficiently anisotropic. Conversely, geometrically anisotropic particles might not produce significant depolarized scattering if their internal composition is otherwise homogeneous. Optical anisotropy is a prerequisite for detecting rotational diffusion, but the rotational and translational diffusion coefficients are determined by particle shape, regardless of whether the particle is internally isotropic or not. This limitation is not specific to our method but is also inherent to DDLS techniques. (3) The physical separation of the orthogonal components via a polarization filter may offer increased robustness across different particle types. Our approach challenges the conventional requirement of always physically separating the two orthogonal polarization (vv and vh) components of scattered light when constructing autocorrelation functions. Instead, we rely on collecting scattered light encompassing all polarization orientations and subsequently apply mathematical or algorithmic methods to disentangle the translational and rotational diffusion modes. Our method provides a flexible alternative, but the characteristic relaxation rates of the translational and rotational modes must be sufficiently distinct to allow for unambiguous separation. If at a given scattering angle the particle size, shape, or associated polydispersity result in significantly overlapping translational and rotational relaxation rates, mathematical separation may become unreliable. In such cases, selecting another more appropriate scattering angle may facilitate clearer mode separation, by benefiting from the angular dependence of the translational diffusion relaxation rate. However, the optimal range of scattering angles is sample-dependent and may require empirical determination. (4) The presence of a clearly bimodal decay in the correlation function is not, by itself, evidence of optical anisotropy. Such bimodality may simply result from a bimodal distribution of translational diffusion of a dual population. To confirm the presence of rotational diffusion, measurements at a minimum of two different scattering angles are required to determine whether the observed mode is independent of the scattering angle. If only one angle is available, prior knowledge of the sample’s properties may be necessary to assess whether the observed mode can indeed be attributed to rotational diffusion.

## Supplementary Material



## Data Availability

All data supporting the findings are available from the corresponding author upon request.

## Notes

Transmission electron microscopy images of the gold nanostars, hematite spindles, and gold nanorods; measured particle sizes and aspect ratios; schematic of the DLS setup; hydrodynamic models and equations for translational and rotational diffusion coefficients; procedure for extracting aspect ratio and particle length from translational and rotational diffusion coefficients (PDF)
