# Addition to “Polarization-Multiplexed Dynamic Light Scattering: Characterizing Rotational Diffusion and Shape of Optically Anisotropic Particles”

**DOI:** 10.1021/acs.analchem.6c04077

**Published:** 2026-07-13

**Authors:** Lukas Grabenwarter, Miguel Spuch-Calvar, Patricia Taladriz-Blanco, Christian Moitzi, Sandor Balog

A disclosure statement was inadvertently omitted from the original manuscript. It is presented below, as it should have appeared in the original publication.

The authors declare the following competing financial interest(s): Sandor Balog discloses a consultancy relationship with Anton Paar GmbH. This relationship had no influence on the design, results, or interpretation of this study.

