# Supplementary material for: Polarization-Multiplexed Dynamic Light Scattering: Characterizing Rotational Diffusion and Shape of Optically Anisotropic Particles
Source: Anal Chem. 2026 Apr 2;98(14):10302–8. doi: 10.1021/acs.analchem.5c08209 (PMC13084620; doi:10.1021/acs.analchem.5c08209)
Supplement: Supplementary file 1 [file ac5c08209_si_001.pdf]

Polarization-multiplexed dynamic light scattering: Characterising rotational diffusion and shape of optically anisotropic particles

Supporting Information

Lukas Grabenwarter,[a] Miguel Spuch-Calvar, [b] Patricia Taladriz-Blanco, [c] Christian Moitzi[a] and Sandor Balog*[c,d]

[a]Anton Paar GmbH, Anton-Paar-Str. 20, 8054 Graz, Austria

[b]CINBIO, Universidade de Vigo - Campus Universitario Lagoas Marcosende, 36310 Vigo, Spain.

[c]Adolphe Merkle, University of Fribourg, 1700 Fribourg, Switzerland

[d]National Center of Competence in Research Bio-Inspired Materials, University of Fribourg, 1700 Fribourg, Switzerland

E-mail: sandor.balog@unifr.ch

Table of Contents

1. **Particles**
   - Gold Nanostars (GNS) – synthesis protocol and purification
   - Hematite Spindles (HES) – synthesis protocol
   - Gold Nanorods (GNR) – commercial source and preparation
   - Materials used
   - Electron microscopy method
   - TEM images and particle dimensions
     - Figure SI-1: TEM images of gold nanostars with Feret diameter distribution
     - Figure SI-2: TEM images of hematite spindles with measured length, width, and aspect ratio
     - Figure SI-3: TEM images of gold nanorods (from Certificate of Analysis) with reported dimensions

2. **DLS layout**
   - Figure SI-4: Schematic of the three-angle DLS instrument and description of the detection scheme

3. **Hydrodynamic models**
   - Diffusion coefficient definitions (DT, DR)
   - Spherical model for estimating hydrodynamic diameter of gold nanostars
   - Axisymmetric models for hematite spindles and gold nanorods
     - Prolate spheroid model
     - Cylinder model (Ortega & García de la Torre)
     - Sphero-cylinder model (Fujita)
   - Procedure to extract aspect ratio (p) and particle length (L) from diffusion data

1. Particles

Gold nanostars (GNS): Gold nanostars were synthesized following the protocol reported by Kumar et al. [2] In brief, 22.4 µL of gold salt solution ([Au] = 111 mM) was added to a solution containing 500 mg of polyvinylpyrrolidone (PVP, Mw = 10,000; TCI Chemicals) previously dissolved in 5 mL of N,N-dimethylformamide (DMF) under magnetic stirring. After two minutes, 33 µL of 15 nm PVP-coated gold nanoparticles ([Au] = 3.5 mM) dispersed in ethanol were added to the mixture. The dispersion was stirred for an additional 15 minutes. Excess PVP was removed by centrifugation at 4500 rpm for 30 minutes, and the purified nanostars were resuspended in Milli-Q water. The gold nanoparticles with an average diameter of 15 nm were prepared using the classical Turkevich method [3] Specifically, 1.82 mL of sodium citrate solution ([NaCit] = 0.47 M) was added in a single step to 200 mL of a boiling aqueous solution of gold salt ([Au] = 0.5 mM) under continuous magnetic stirring. Immediately after citrate addition, the heating source was turned off, and the dispersion was maintained under stirring for 15 minutes. The suspension was then cooled to room temperature and stored at 4 °C until further use. To coat the 15 nm gold nanoparticles with PVP, 200 mL of the as-prepared gold seeds ([Au] = 0.5 mM) were mixed with 5 mL of an aqueous PVP solution ([PVP] = 60 mg/mL; Mw = 10,000; TCI Chemicals). The mixture was stirred for 24 hours. Excess PVP was removed by centrifugation at 7000 rpm for 1 hour, and the coated particles were resuspended in ethanol.

Hematite spindles (HES): Hematite spindle-shaped particles were synthesized following the approach described elsewhere.[1] An aqueous solution containing 0.02 M $FeCl_3$ and 4.5×10^{-4} M $NaHPO_4$ was placed in an oven at 100 °C for 48 hours. The particles were washed three times with deionized water and subsequently redispersed in Milli-Q water.

Materials used: Gold (III) chloride trihydrate ($HAuCl_4 \cdot 3H_2O$, ≥99.9%), sodium citrate tribasic dihydrate ($C_6H_5Na_3O_7 \cdot 2H_2O$, ≥98%), and N,N-dimethylformamide (DMF, anhydrous, 99.8%), Iron(III) chloride ($FeCl_3$, 97%), and sodium hydrogen phosphate ($NaHPO_4$) (Sigma-Aldrich). Polyvinylpyrrolidone (PVP, $(C_6H_9NO)_n$, M_w = 10k, TCI chemicals). Milli-Q grade water was used for all preparations.

Electron microscopy: The particles were visualized by transmission electron microscopy (TEM) using an FEI Tecnai Spirit TEM (ThermoFisher, Waltham, MA, USA) operating at 120 kV. NPs were deposited onto a carbon film-coated 300-mesh copper grid (Electron Microscopy Sciences, Pennsylvania, PA, USA) and dried at room temperature before imaging. Bar inclusion and size distribution (manual counting) were conducted using Fiji (ImageJ version 1.53).

Gold nanorods (GNR): Citrate coated gold nanorods were purchased from nanoComposix (NanoXact, product number: GRCN660, Lot number: JLC0070). They were subjected to gentle centrifugation (3000 rpm for 10 min) prior to measurement.

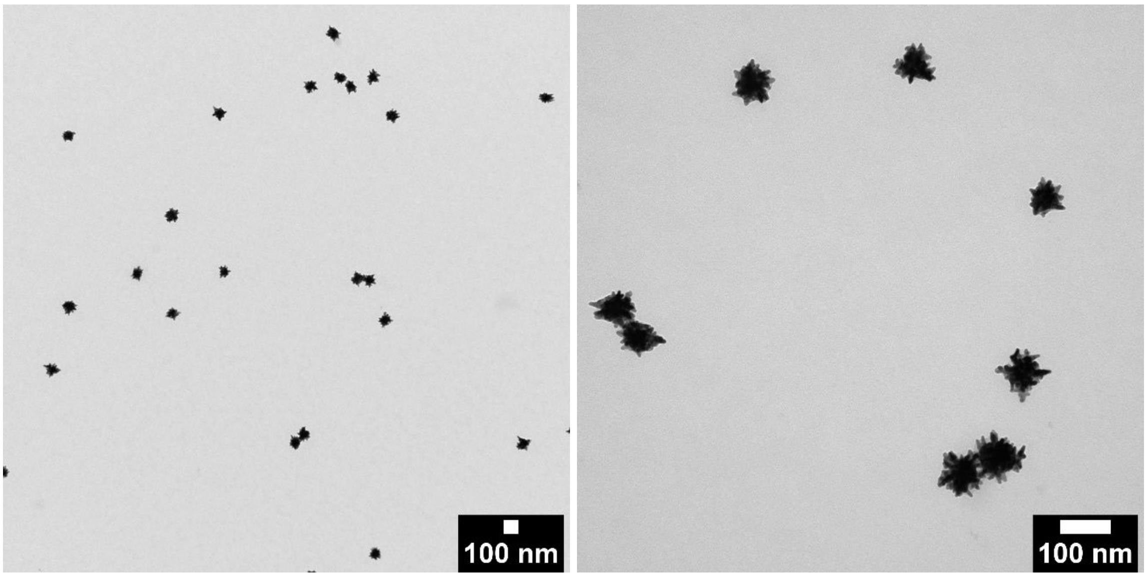

Figure SI-1. Transmission electron micrographs of gold nanostar particles. The spiky, round particles exhibit a Feret diameter of 96±9.5 nm (mean±STD).

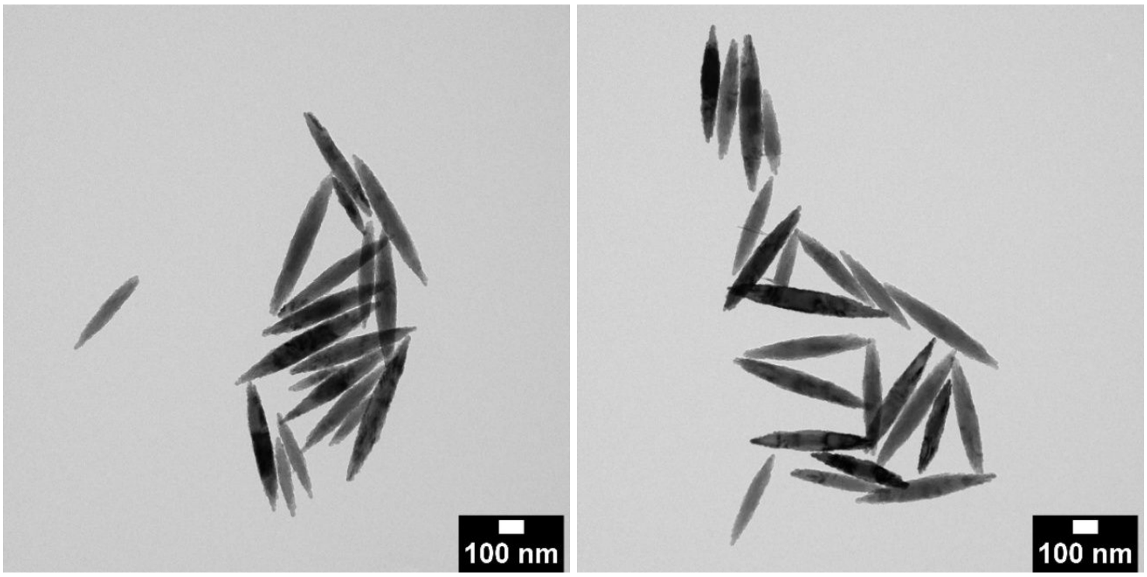

Figure SI-2. Transmission electron micrographs of HES particles. These spheroid-like particles exhibited a length of (482±100) nm, a width of (74±13) nm, and aspect ratio of 6.5±0.7**.**

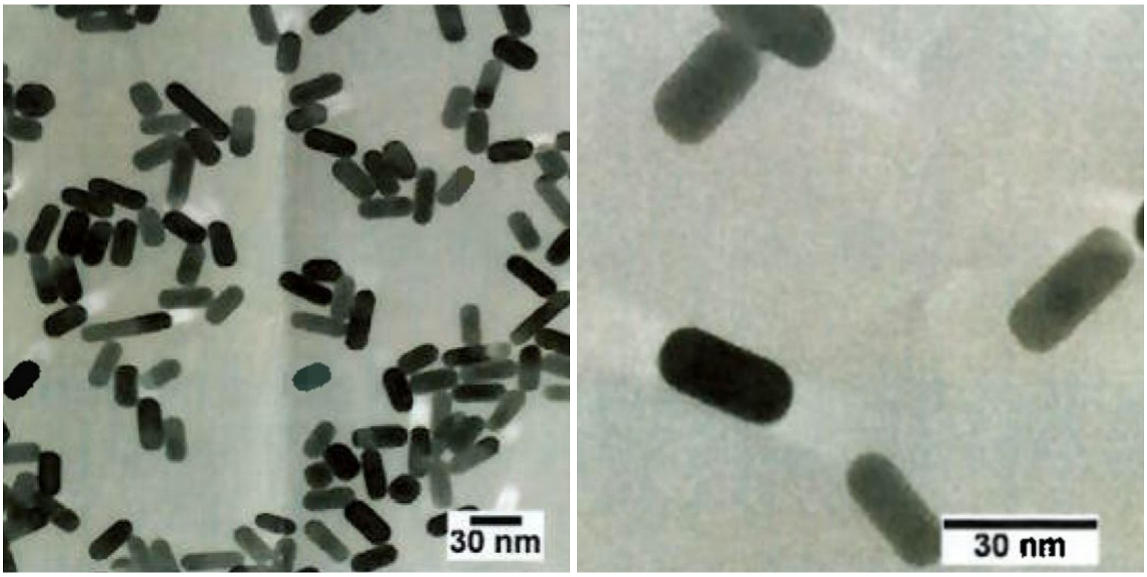

Figure SI-3. Transmission electron micrographs of GNR particles. (Copied from the Certificate of Analysis) According to the certificate, these particles exhibited a length of (32±5.5) nm, a width of (13±1.5) nm, and a mean aspect ratio of 2.5 (no information given on STD).

2. DLS layout

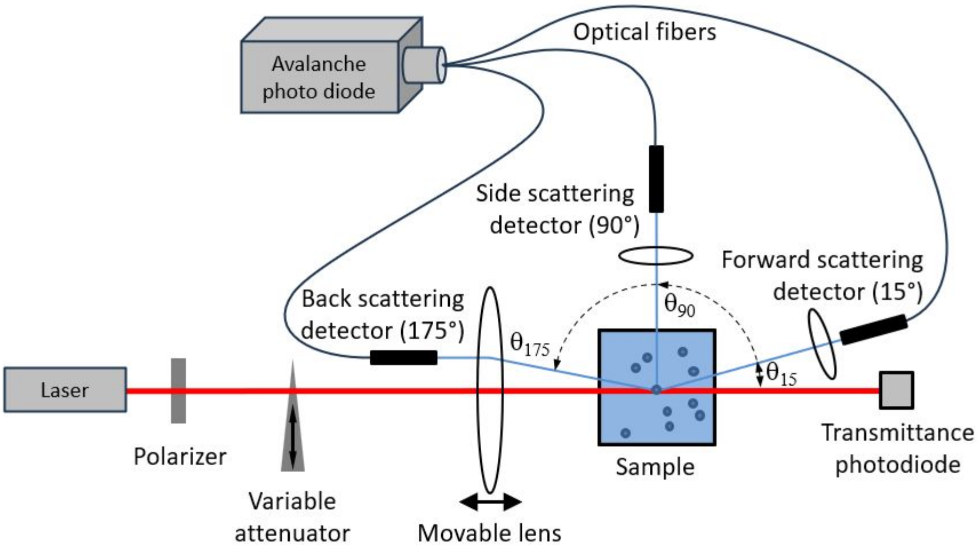

Figure SI-4: Schematic representation of the three-angle dynamic light scattering (DLS) instrument used for the analysis of anisotropic particles. A vertically polarized laser beam (wavelength λ=658 nm, 20 mW) passes through a vertical polarizer and a variable attenuator to define the incident polarization state and intensity. The laser beam is focused into the sample cell by a movable lens. Particles undergoing translational and rotational Brownian motion scatter the incident light. Scattered

radiation can be collected at selectable scattering angles θ = 15° (forward scattering), θ = 90° (side scattering), and θ = 175° (backscattering). The collected light from each angle is coupled into optical fibers that are connected to a single avalanche photodiode (APD) detector with an optimal photon count-rate between 200 and 300 kHz. Shutters (not shown) are used to select one fiber at a time, such that measurements may be performed sequentially at different scattering angles. No polarization optics are used in the detection path, so the detector collects all polarization components simultaneously. The measured signal is therefore a mixture of linear compositions of vertically polarized (vv) and depolarized (vh) contributions. As a result, the intensity autocorrelation function contains two types of relaxation: a scattering-angle-dependent relaxation from translational diffusion, and a scattering-angle-independent relaxation from rotational diffusion.

3. Hydrodynamic models

The translational diffusion coefficient is given by $D_T = \Gamma_1/q^2$, and the rotational diffusion coefficient by $D_R = \Gamma_2/6$. These expressions are provided here for completeness.

The hydrodynamic diameter of gold nanostars (GNS) were estimated via the model of spheres, by inverting the translational and rotational expression: $\frac{2\,\kappa}{6\,D_T}$ and $\left(\frac{8\,\kappa}{8\,D_R}\right)^{1/3}$, where D_T is the translation diffusion coefficient, D_R rotational diffusion coefficient, and $\kappa = k_B T/\pi\,\eta$ at temperature T and viscosity η.

The gold nanorods (GNR) and hematite spindles (HES) were modelled as axisymmetric particles, with length L and aspect ratio p. For HES the model of prolate spheroids were used

1) $D_T = \frac{\kappa}{3L}\frac{p}{\sqrt{p^2-1}}\mathrm{Log}[p + \sqrt{p^2-1}]$
2) $D_R = \frac{\kappa}{(2/3)L^3}\frac{(2p^2-1)\,\mathrm{Log}[p+\sqrt{p^2-1}]\Big/\left(p\sqrt{p^2-1}\right)-1}{(p^4-1)/p^4}$.

For gold nanorods, we used two models, which may be expressed as

1) $D_T = \frac{\kappa}{3L}(\mathrm{Ln}\,p + f_T)$
2) $D_R = \frac{3\,\kappa}{L^3}(\mathrm{Ln}\,p + f_R)$

where $f_T$and f_R are model-dependent functions of the aspect ratio.

The Ortega & García de la Torre model of cylinders gives:

1) $f_T = 0.312 + \frac{0.565}{p} - \frac{0.1}{p^2}$
2) $f_R = -0.662 + \frac{0.917}{p} - \frac{0.05}{p^2}$,

and the Fujita model of sphero-cylinders gives

1) $f_T = 0.3863 + \frac{0.0005859}{p^5} - \frac{0.000651}{p^4} - \frac{0.01042}{p^3} - \frac{0.0625}{p^2} + \frac{0.6863}{p}$
2) $f_R = -\frac{11}{6} - \frac{33.27076}{p^{3/2}} + \frac{140.26992}{p^{5/4}} - \frac{218.8365}{p} + \frac{174.0921}{p^{3/4}} - \frac{62.6084}{\sqrt{p}} + \frac{13.0447}{p^{1/4}} + \mathrm{Ln}\,4 - \frac{8.256}{\mathrm{Ln}(1+p)}$

To determine length L and aspect ratio p of HES and GNR, we used the following steps: 1) We recognized that the expression $\gamma = \frac{D_R}{{D_T}^3}\kappa^2$ is dimensionless and depends only on the aspect ratio p. Therefore, we first used the function $\gamma(p)$to obtain p. Once p was known, we substituted it into the expression for D_T (or equivalently D_R) to solve for the particle length L.

References

[1] Spuch-Calvar, Pérez-Juste, et al., Hematite spindles with optical functionalities: Growth of gold nanoshells and assembly of gold nanorods, J. Colloid Interface Sci. 310 (2007) 297–301

[2] Kumar, Pastoriza-Santos, et al., High-yield synthesis and optical response of gold nanostars, Nanotechnol. 19 (2008) 15606

[3] Enüstün, Turkevich, Coagulation of Colloidal Gold, J. Am. Chem. Soc. 85 (1963) 3317
